# Propagation of spontaneous electrical activity in the ex vivo human uterus

**DOI:** 10.1007/s00424-020-02426-w

**Published:** 2020-07-20

**Authors:** Nienke P.M. Kuijsters, Federica Sammali, Xin Ye, Celine Blank, Lin Xu, Massimo Mischi, Benedictus C. Schoot, Chiara Rabotti

**Affiliations:** 1 Department of Electrical Engineering (Signal Processing Systems: Biomedical Diagnostics), Eindhoven Technical University, Post box 513, 5600 MB Eindhoven, the Netherlands; 2 Department of Obstetrics and Gynaecology, Catharina Hospital, Michelangelolaan 2, 5623 EJ Eindhoven, the Netherlands; 3 Department of Obstetrics and Gynaecology, University Hospital (UZ) Gent, C. Heymanslaan 10, 9000 Ghent, Belgium; 4 School of Information Science and Technology, ShanghaiTech University, Shanghai, China

**Keywords:** Uterine electrophysiology, Uterine contractility, Smooth muscle, Propagation, Pacemaker, Fertility

## Abstract

Contractions of the non-pregnant uterus play a key role in fertility. Yet, the electrophysiology underlying these contractions is poorly understood. In this paper, we investigate the presence of uterine electrical activity and characterize its propagation in unstimulated ex vivo human uteri. Multichannel electrohysterographic measurements were performed in five freshly resected human uteri starting immediately after hysterectomy. Using an electrode grid externally and an electrode array internally, measurements were performed up to 24 h after hysterectomy and compared with control. Up to 2 h after hysterectomy, we measured biopotentials in all included uteri. The median root mean squared (RMS) values of the external measurements ranged between 3.95 μV (interquartile range (IQR) 2.41–14.18 μV) and 39.4 μV (interquartile range (IQR) 10.84–105.64 μV) and were all significantly higher than control (median RMS of 1.69 μV, IQR 1.13–3.11 μV), consisting of chicken breast meat. The RMS values decreased significantly over time. After 24 h, the median RMS (1.27 μV, IQR 0.86–3.04 μV) was comparable with the control (1.69 μV, IQR 1.13–3.11 μV, *p* = 0.125). The internal measurements showed a comparable pattern over time, but overall lower amplitude. The measured biopotentials propagated over the uterine surface, following both a plane-wave as well as an erratic pattern. No clear pacemaker location nor a preferred propagation direction could be identified. These results show that ex vivo uteri can spontaneously generate propagating biopotentials and provide novel insight contributing to improving our understanding of the electrophysiology of the human non-pregnant uterus.

**Electronic supplementary material:**

The online version of this article (10.1007/s00424-020-02426-w) contains supplementary material, which is available to authorized users.

## Introduction

Similar to other smooth muscle organs, the uterus is able to spontaneously contract. While the contractile activity of the uterus is mostly known for its role in the expulsion of the foetus during labour at the end of pregnancy, it also contracts throughout the menstrual cycle of women in their fertile age [22]. Inspired by ultrasonographic observations, during the menstrual cycle, uterine activity is mostly referred to as uterine peristalsis.

There is strong evidence that uterine peristalsis plays a role in fertility, either positive, providing transportation of gametes [23], or negative, by interfering with proper implantation of the embryo [11]. The function of uterine peristalsis has therefore been investigated to understand its role in fertility treatments and possibly develop techniques to monitor or even modulate uterine peristalsis [11, 12, 16, 52]. However, fundamental aspects of the physiology underlying uterine peristalsis remain unclear.

Smooth muscle contractions are invariably initiated by the presence of biopotentials propagating at the cell level. The uterus is provided with sympathetic and parasympathetic innervation. Nevertheless, previous studies demonstrated the ability of the uterus to initiate contractions spontaneously, even when disconnected from the nervous system [3, 44]. This suggests that uterine electrical activity is initiated by an intrinsic source with its own pace-making units. It is hypothesized that specialized cells found in the myometrium of both animals and humans, called interstitial Cajal-like cells (ICLCs), are involved in this self-initiating mechanism [10], though up to now, their role remains unsettled. A thorough characterization of the generation and propagation of biopotentials in the non-pregnant uterus may be crucial to advance the basic knowledge of uterine peristalsis and possibly unveil the great potential of contraction management for improving fertility.

In general, studies on the electrical activity of the non-pregnant human uterus are scarce [3, 43, 45]. On the contrary, significant advances have been reported on the characterization of uterine electrical activity in pregnant uteri by abdominal electrohysterography (EHG) [26, 27, 29, 38, 48, 51]. All these studies, aiming at improving labour monitoring and preterm delivery prediction, report patterns of cyclic bursts of biopotentials alternating, in time, with quiescent periods [37, 47]. Spatially, the results of dedicated investigations exclude the existence of a preferred origin and direction of biopotential propagation, and support the validity of both erratic and plane-wave propagation patterns [35].

Clearly, a direct translation of the observations derived during pregnancy to the non-pregnant uterus is unfeasible. Moreover, in vivo characterization of the biopotentials of the non-pregnant human uterus by EHG is severely hampered by challenges related, for example, to the small size of the uterus, the distance between the external electrodes and the uterus, and the interference of other organs. These anatomical constraints contribute to a deterioration of the signal-to-noise ratio, complicating the propagation analysis of such unpredictable signals as uterine biopotentials.

Ex vivo investigations on the intact organ combined with the use of multichannel measurement of the biopotentials are expected to overcome the limitations imposed by in vivo measurements while substantially advancing current understanding of biopotential propagation in the non-pregnant uterus.

Previous ex vivo studies evaluating contractions on the complete non-pregnant human uterus used extracorporeal perfusion to test the effect of pharmaceutics on uterine contractility and are mainly based on intrauterine pressure measurements [3, 39, 40, 45]. Attempts at measuring biopotentials can be found in two studies [3, 45], in which observations are limited to a single location in the uterus. Only one study eventually measured biopotentials using a single needle placed at the utero-tubal junction [45], while the other could not measure any electrical activity using 2 bipolar Ag/AgCl electrodes inserted into the uterine wall [3]. No literature is available on the propagation of biopotentials in the non-pregnant human uterus.

In this observational study, we investigated spontaneous biopotentials of the non-pregnant human uterus ex vivo and analyzed both qualitative and quantitative aspects of the biopotentials’ propagation, using multichannel EHG measurements. In order to detect the presence of spontaneous biopotentials, both externally and internally, we evaluated the root mean squared (RMS) values of the EHG signal of five resected uteri and compared them with control. Furthermore, we investigated the propagation patterns of the EHG signal. In particular, we analyzed the EHG propagation velocity by a validated maximum likelihood approach [34].

Since a significant decay of the measured biopotentials can be expected as a consequence of progressive tissue deterioration, the RMS values were also evaluated over a time span of 24 h, starting immediately after hysterectomy.

## Methods

### Subjects

Five human uteri were included in this study. Patients were eligible if they were prescribed to undergo a hysterectomy for heavy menstrual bleeding (hypermenorrhoea) or painful menstruations (dysmenorrhoea), and were all premenopausal. Standard laparoscopic hysterectomy procedures were performed on all uteri. Exclusion criteria included any risk of malignancy and any upfront diagnosed uterine pathologies such as big leiomyomas or adenomyosis, which might interfere with the measurements. The day of the menstrual cycle was recorded by anamnestic means.

### Measurement set-up and data acquisition

Directly after resection (approximately 20–30 min after eliminating blood supply), the uteri were incorporated into the measurement set-up. Room temperature was kept constant at 21 °C. No preservative measures were used for the resected organs. All uteri were measured directly after surgical removal (*T0*, 30-min duration), 1 h after removal (*T1*, 15-min duration), and 2 h after removal (*T2*, 15-min duration). One patient (patient 1) had an additional measurement 24 h after removal (*T24*, 15-min duration).

**Fig. 1:**
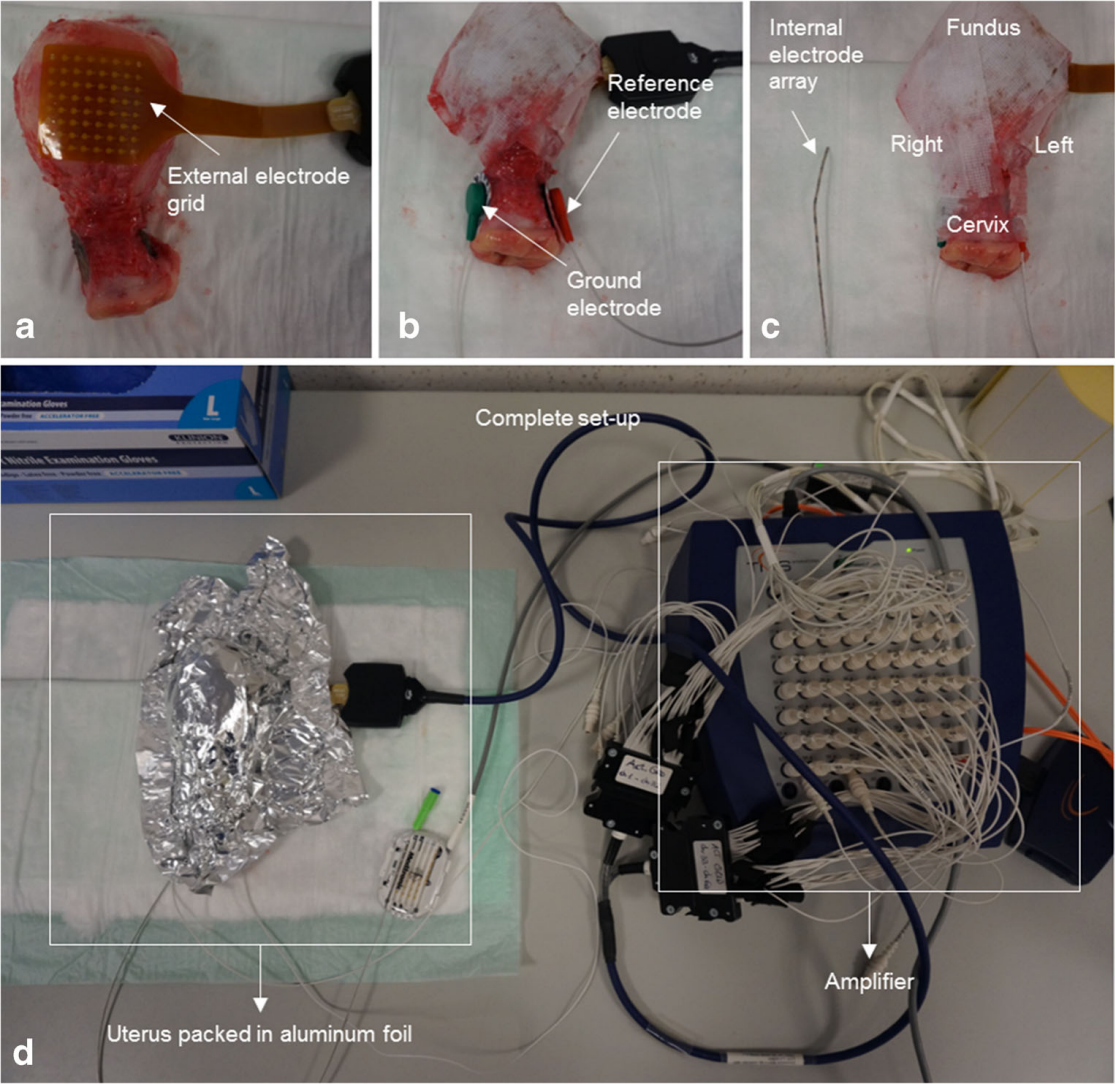
Measurement set-up. The external grid is placed directly on the surface of the ex vivo uterus and fixed using paper-based tape. **a**. Fifty-five channels of the grid are used for the analysis. **b**. Two silver-based surface electrodes, representing the ground and reference electrode, are placed on the left and right side of the uterine cervix and fixed with paper-based tape. **c**. The internal array with eight electrodes (shown next to the uterus for scale) is inserted into the uterine cavity. **d**. All electrodes are connected to the amplifier for simultaneous recording and the uterus is wrapped in aluminium foil to keep moisture and avoid any deterioration of the electrical contact. Take note: In order to avoid delays in the measurements, the pictures were taken after the experiment. This explains also the imprint of the silver electrodes which is visible in picture A

EHG measurements were simultaneously performed externally on the uterine surface using a high-density grid and inside the uterine cavity by an array of eight platinum electrodes mounted on a silicon catheter (Fig. 1). As a control, identical measurements were performed on chicken breast meat (commercial nutritional use) under the assumption that no significant biopotentials could be measured on this muscle.

Two-dimensional electrode grids were needed for a complete characterization of the propagating waves, as the a priori origin and direction were unknown. We placed a high-density 64-electrode grid (TMSi, Oldenzaal, the Netherlands) directly on the external surface of the resected organ. Each of its circular Ag/AgCl electrodes (2-mm diameter) is printed on the flexible grid following a regular, two-dimensional pattern with 4-mm centre-to-centre inter-electrode distance, covering a total 36 × 36-mm^2^ area. The grid was positioned in the middle of the uterine body and held in place using a medical tape. No adhesive sticker or conductive gel was employed as the uterine natural moisture functioned as a conductive layer. After sensor placement, the uterus was wrapped in aluminium foil to avoid evaporation and deterioration of the electrical contact of the grid with the tissue.

For internal measurements, a polyurethane octapolar electrode array (Pisces Plus lead model 3877, Medtronic, Minneapolis, MN, USA), normally used for spinal cord stimulation, was positioned inside the uterine cavity. This cylindrical (diameter = 1.3 mm) electrode array contains eight 6-mm platinum electrodes, with an inter-electrode distance of 12 mm and a total array length of 132 mm. Two circular 24-mm disposable surface EMG/ECG electrodes (Covidien, part of Medtronic, Minneapolis, MN, USA) were secured on the cervix with circular tape (Fig. 1) and used as ground and reference, since electrical propagation in the uterus is most commonly measured using unipolar measurements [35]. The cervix was chosen as reference because it is assumed to be the most electrically neutral, as it does contain the least smooth muscle tissue [46]. Once all the electrodes were in place and the set-up was ready for the measurement, we maintained this set-up in the same condition without any alteration up until the end of the last measurement.

All biopotentials were simultaneously recorded and digitized at 1024-Hz sampling frequency using a Refa 72 Amplifier (TMSi, Oldenzaal, Netherlands), connected to a laptop with the PolyBench 5 software (TMSi, Oldenzaal, Netherlands) for real-time visualization of the signals. Signal analysis was performed off-line using MATLAB (The MathWorks^®^ Inc., Natick, MA, USA).

### Pre-processing

The acquired signals were first made unipolar by subtracting the common reference signal and then down-sampled from 1024 to 32 Hz after proper antialiasing filtering. In vivo EHG studies commonly use a filter range from 0.34 to 1 Hz in order to avoid interference from signals produced by e.g. cardio-respiratory activity [13, 30]. In this study, an upper frequency limit of 5 Hz was chosen, as we expected no interferences and higher-frequency components when measuring directly on the uterus [36]. For similar reasons, due to the absence of respiration artefacts and the low frequency content of EHG signals, a lower frequency limit of 0.05 Hz was used. The filter was implemented as a cascade of a low-pass and a high-pass 6th-order Butterworth filters.

### Signal amplitude estimation

Amplitude estimators such as the RMS value have been previously used to derive an estimate of EHG signal amplitude [17, 41]. The RMS value RMS_*c*_ of the discrete signal *x*_*c*_[*n*] recorded by channel *c* is calculated as follows:

1 $$ {\mathrm{RMS}}_c=\sqrt{\frac{1}{N}{\sum}_{n=1}^N{\left|{x}_c\left[n\right]\right|}^2}\kern0.5em $$

where *x*_*c*_[*n*] is the down-sampled, filtered signal obtained from one single electrode of the grid and *N* is the total number of time samples considered.

In this study, the RMS value was used as feature for two different objectives: to evaluate the signal amplitude of the recorded uteri in the first hour after removal as compared with control muscle (chicken meat) and to quantify the signal amplitude decay over time after surgical removal. In both cases, median and interquartile range of the RMS values of the signal in the external grid were used as features.

### Propagation detection

Under the assumption that propagating biopotentials are recorded as similar, delayed spikes at different locations, coherence was used as similarity measure to detect propagation. At the cell level, uterine biopotentials have been reported to occur in groups (bursts) [31, 37, 47]. However, propagation analysis of individual electrical spikes, i.e. the surface signal peaks associated to individual action potentials, has been proven more informative [35, 38]. Both for the external grid and for the internal catheter, propagation analysis was therefore based on individual spikes selected by 50-s sliding windows (25-s overlap). Preliminary inspection of our data suggested such a time window to be optimal to isolate single spikes.

Based on the segmented signals, the first step aimed at identifying events of propagating spikes on the external grid. We identified propagation with the detection of spikes measured by *N*_*P*_ contiguous channels with a similar shape and a certain delay. In formulas, considering in the current 50-s time interval, the pre-processed discrete signals *x*_*C*1_(*n*), *x*_*C*2_(*n*), …${x}_{C{N}_P}(n)$ (*n* ∈ [1, 2, ⋯*N*]) in the contiguous channels (by row, by column, or diagonally) *C*1, *C*2…,*CN*_*P*_, respectively, are assumed to propagate when they satisfy the equations:

2 $$ {x}_{C1}(n)=s\left(n+{\tau}_1\right)+{w}_1(n){x}_{C2},(n)=s\left(n+{\tau}_2\right)+{w}_2(n),{x}_{C{N}_P}(n)=s\left(n+{\tau}_{N_P}\right)+{w}_{N_P}(n). $$

In (2), the same noise-free shape *s*(*n*) is recorded by electrodes *C*1, *C*2,… *CN*_*P*_ with delays *τ*_1_, *τ*_2_, and ${\tau}_{N_P}$ after the addition of white Gaussian noise *w*_1_(*n*), *w*_2_(*n*), and ${w}_{N_P}(n)$.

**Fig. 2:**
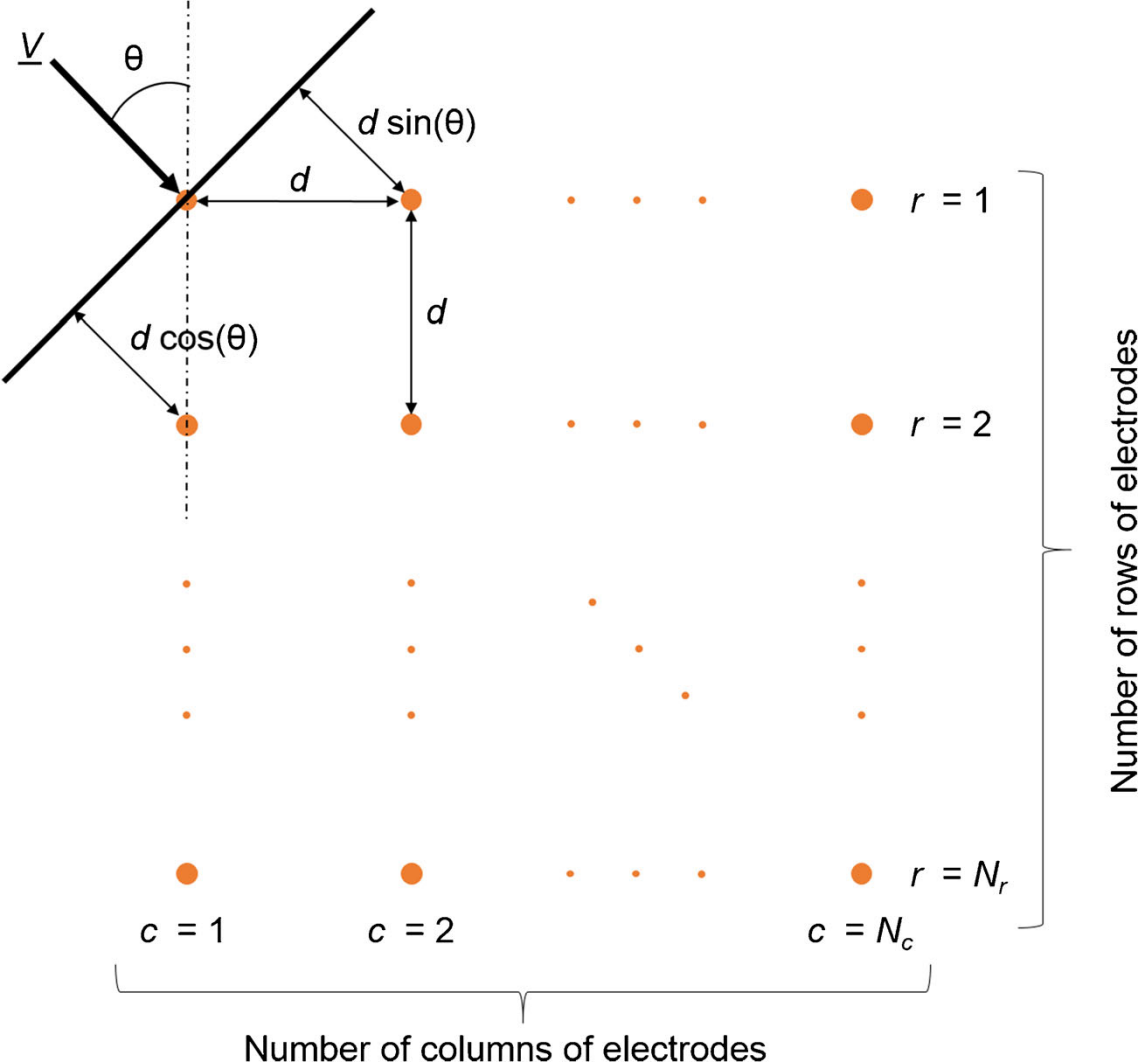
Schematic description of plane-wave propagation

Considering the noise-free biopotential *s*(*n*) propagating through a set of electrodes arranged in a grid composed of *N*_*r*_ rows and *N*_*C*_ columns (Fig. 2), the plane-wave condition is satisfied when the measured signal *x*_*rc*_(*n*) at the channel (*r*, *c*) in the *r*^th^ row (*r* ∈ [1, 2, ⋯, *N*_*r*_]) and *c*^th^ column (*r* ∈ [1, 2, ⋯, *N*_*c*_]) can be modelled as:

3 $$ {x}_{rc}(n)=s\left(n-\left(r-1\right){\tau}_r-\left(c-1\right){\tau}_c\right)+{w}_{rc}(n), $$

i.e. the delay between adjacent rows and columns of electrodes, τ_r_ and τ_c_ , respectively, is constant throughout the electrode set. The noise present in the signal recorded by channel (*r*, *c*), *w*_*rc*_(*n*), is assumed to be white and Gaussian with variance ${\upsigma}_{rc}^2$.

The aim of this first step was to register both the time segments and the electrode subset *CN*_*P*_ for which the propagation condition described in (2) could be considered valid on the external grid. To this end, coherence was chosen as the similarity feature.

Given two signals *x*_*C*1_(*n*) and *x*_*C*2_(*n*), the magnitude squared coherence is a real-valued function defined in the frequency domain, *f*, as

4 $$ {C}_{x_{C1}{x}_{C2}}(f)=\frac{\left|{G}_{x_{C1}{x}_{C2}}{(f)}^2\right|}{G_{x_{C1}{x}_{C1}}(f){G}_{x_{C2}{x}_{C2}}(f)}, $$

where ${G}_{X_{C1}{X}_{C2}}(f)$ is the cross-spectral density between *x*_*C*1_ and *x*_*C*2_, and ${G}_{{\mathrm{x}}_{\mathrm{C}1}{\mathrm{x}}_{\mathrm{C}1}}(f)$ and ${G}_{x_{C2}{x}_{C2}}(f)$ the power spectral densities of *x*_*C*1_ and *x*_*C*2_, respectively. The coherence function estimates the extent to which *x*_*C*2_(*n*) may be predicted from *x*_*C*1_(*n*) by an optimum linear least squares function. The magnitude squared coherence is not affected by time delays and reflects only the similarity between signal shapes. Values of coherence will always satisfy $0\le {C}_{X_{C1}{X}_{C2}}(f)\le 1.$

For each 50-s time window, coherence was calculated among all available combinations of signal couples *x*_*C*1_(*n*) and *x*_*C*2_(*n*) using a Hamming window such that each signal was divided into eight overlapping (50%) segments. The maximum value of the coherence function in the frequency range between 0.05 and 5 Hz, which is the frequency band of interest given by the pre-processing filters, was used as similarity feature.

A value of maximum coherence equal to 0.75 was set as threshold to select the signals satisfying the condition in (2). Eventually, only time segments in which one or more channels had a high coherence with at least two other channels in different rows or columns were selected as propagating events. In fact, due to the a priori unknown origin and direction of uterine biopotentials, a plane (3 points) is necessary for identification of the EHG two-dimensional velocity vector. Those selected channels and time segments were then chosen for further analysis of the conduction velocity.

For biopotentials showing plane-wave propagation, i.e. satisfying (3), we searched for propagation in the internal array. Also in this case, the maximum of the coherence function and a value equal to 0.75 were chosen as the similarity feature and threshold, respectively. The internal and external velocity vectors identified according to the approach described in the following paragraph were then compared in the direction parallel to the internal array.

### Conduction velocity

Visual analysis of the propagating segments revealed the presence of both events with plane-wave propagation, i.e. a wave front with a constant direction and speed as in (3), as well as erratic patterns characterized by re-entries and loops [25]. Characterization of propagation was carried out separately, depending on the validity of the plane-wave propagation conditions. For plane-wave propagation events, speed and direction of the conduction velocity vector were estimated using a maximum likelihood approach previously proposed for EHG analysis in pregnancy [34].

Based on the plane-wave condition in (3), *τ*_*r*_ and *τ*_*c*_ can be estimated according to a maximum likelihood approach and therefore by minimizing the cost function [1]:

5 $$ {\epsilon}^2\left({\tau}_r,{\tau}_c\ \right)={\sum}_{r=1}^{N_r}{\sum}_{c=1}^{N_c}{\sum}_{n=1}^N{\left[{x}_{rc}(n)-s\left(n-\left(r-1\right){\tau}_r-\left(c-1\right){\tau}_c\right)\right]}^2 $$

By using Parseval’s equality, *ϵ*^2^ can be transformed into the frequency domain, where *τ*_*r*_ and *τ*_*c*_ become continuous multiplicative factors of the phase and can be estimated without resolution limits.

Furthermore, the propagating biopotential, *s*(*n*), can be estimated in the frequency domain as the weighted average of all channels, *X*_mp_(*f*) in row *m* and column *p*, after alignment. The resulting estimated cost function ${\hat{E}}^2\left({\tau}_{\mathrm{r}},{\tau}_{\mathrm{c}}\ \right)$ is:

6 $$ {\hat{E}}^2\left({\tau}_{\mathrm{r}},{\tau}_{\mathrm{c}}\ \right)=\frac{2}{N}{\sum}_{r=1}^{N_r}{\sum}_{c=1}^{N_c}{\sum}_{f=1}^{N/2}{\left[{\mathrm{X}}_{\mathrm{r}\mathrm{c}}(f)-\frac{1}{N_r{N}_c}{\sum}_{m=1}^{N_r}{\sum}_{p=1}^{N_c}{\mathrm{X}}_{\mathrm{mp}}(f){e}^{j2\pi f\left[\left(m-r\right){\tau}_r+\left(p-c\right){\tau}_c\right]}\right]}^2, $$

where *X*_*rc*_ (*f*) is the discrete Fourier transform (DFT) of the signal recorded by the channel in row *r* and column *c*. For electrode distance equal to *d* and temporal-sampling frequency *f*_*s*_, the speed *v* and angle *θ* can be computed by the relations:

7 $$ {\tau}_r=\frac{f_sd\cos \left(\theta \right)}{v} $$

8 $$ {\tau}_c=\frac{f_sd\sin \left(\theta \right)}{v} $$

### Statistical analysis

In order to assess the statistical significance of our findings, SPSS statistics 23 (SPSS Inc., Chicago, IL, USA), was used. The Shapiro–Wilk test showed that our RMS data, both from the external grid and from the internal array, were not Gaussian distributed. RMS data were presented as the median and the interquartile range. To compare each individual uterus (*n* = 5) with control (*n* = 1), we used a Mann–Whitney *U* test for non-Gaussian data. To assess the significance of the RMS decay in time, we used a generalized linear model for repeated measures [6] comparing the RMS total data of all five uteri at each time (*T*). This model assumes that the input data is Gaussian distributed. By logarithmic transformation of our data, the requirements for a Gaussian distribution (skewness and kurtosis values between − 1 and + 1) were met. To evaluate the correlation between the propagation speed obtained by internal and external recordings, Spearman’s rank correlation coefficient test was used. The level of statistical significance was set at *p* < 0.05 for all tests used.

## Results

### Subjects

In this study, we included five women referred for hysterectomy who were aged between 35 and 44. In four out of five uteri, pathologic examination showed adenomyosis. One patient also had a small leiomyoma. One patient was menstruating at the time of surgery (Table 1).

**Table 1:** Patient characteristics and number of propagation events found per patient

Patient	Age	Pathology found in the uterus	Phase of the cycle	T0			T1	T2
				Erratic propagation events in external grid	Plane-wave propagation events in external grid	Propagation events in internal array	Plane-wave propagation events in external grid	Plane-wave propagation events in external grid
1	44	Leiomyoma Adenomyosis	Day 28, first cycle on progesterone treatment	6	0	6	-	-
2	44	Adenomyosis	Day 1, menstruation	1	17	12	5	3
3	48	Adenomyosis	Irregular cycle	10	1	5	-	-
4	35	Adenomyosis	Day 10, follicular phase	6	0	8	-	-
5	38	None	Day 7, follicular phase	10	0	4	-	-

### Detection of biopotentials

**Fig. 3:**
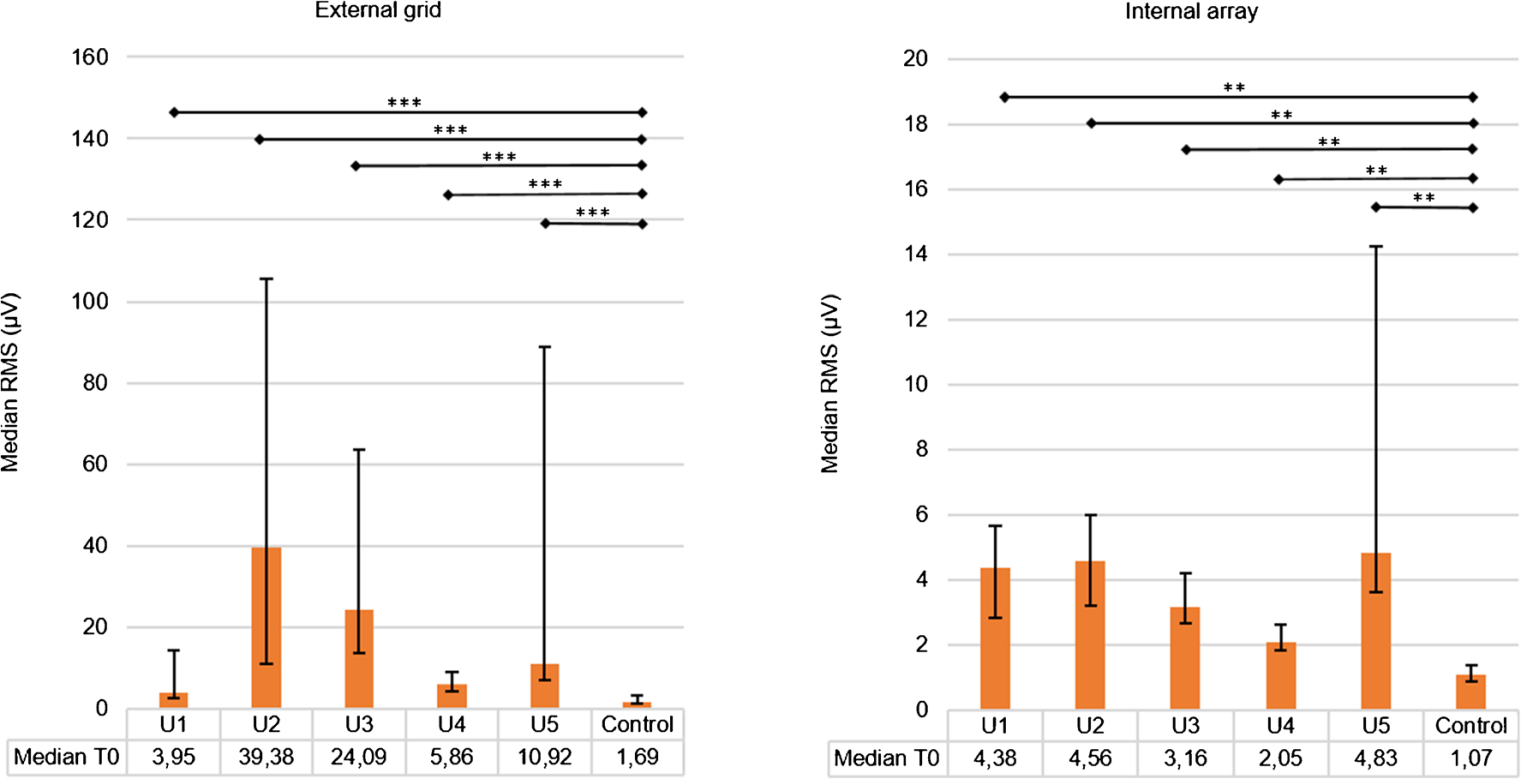
RMS values of each uterus (U1, U2, U3, U4, and U5) and the chicken breast meat (control) measured at T0. The results are expressed as median RMS of the signals of the external grid (55 signals per uterus) and the internal array (8 signals per uterus). The error bars represent the interquartile range. Significant difference with the control, tested with a Mann-Whitney U test (SPSS Inc., Chicago, IL, USA), are indicated by asterisks: *** p value < 0.001 and **p value < 0.01

The presence of electrical activity was detected by estimating the root mean square (RMS) value as indicator of the signal amplitude of the EHG signal recorded at *T0* by the external grid and the internal array. We calculated a median RMS of the 55 external grid channels for each uterus, after excluding the channels with poor contact (maximum 2 per grid). Medians between 3.95 μV (interquartile range (IQR) 2.41–14.18 μV) and 39.4 μV (IQR 10.84–105.64 μV) were obtained for the external grid (Fig. 3). Compared with control (median 1.69 μV, IQR 1.13–3.11 μV), we found a significant difference in RMS values for all five uteri (*p* values < 0.05). The internal array showed median RMS values between 2.05 μV (IQ range 1.84–2.60 μV) and 4.83 μV (IQR 3.62–14.23 μV), which were all significantly different from control (median 1.07 μV, IQR 0.87–1.37 μV) with *p* values < 0.05.

**Fig. 4:**
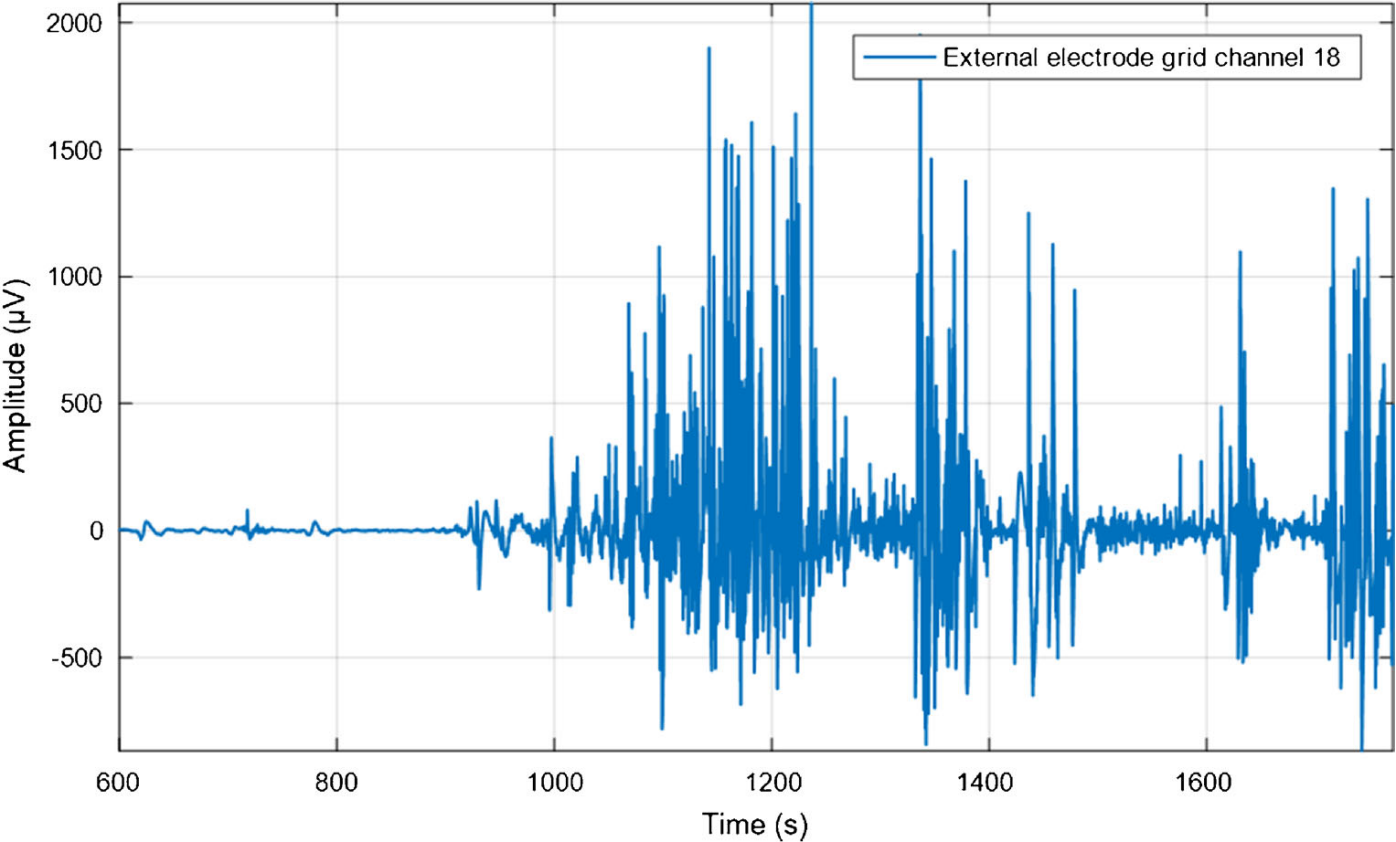
Example of a burst recorded from channel 18 of the external grid on uterus number two at T0

Qualitatively, visual inspection of the individual channels revealed isolated spikes of biopotentials rather than clear bursts, with the exception of uterus number 2, which showed distinct bursts (Fig. 4).

### Characterization of biopotential propagation

Propagation was detected in all five uteri (Table 1). A median of 10 propagation events (range 6–18) was observed per 30-min measurement. In line with previous studies in pregnancy [35], visual analysis of the propagating segments revealed the presence of both plane-wave propagation, i.e. wave fronts with a constant direction and velocity, and different events showing spikes with an erratic pattern, characterized by re-entries and circulation [25]. Examples of plane-wave and erratic propagation are shown in Fig. 5. In total, 18 plane-wave propagation events were detected. Noteworthy, 17 out of 18 were detected in uterus number 2 and this uterus did not show any erratic propagation. No deviation in age, body mass index, use of medication, or operating time was found in patient number 2 relative to the other patients in the dataset. The only singularity of patient number 2 was that she was the only woman in the menses phase at the time of surgery. A total of 33 events of detected propagating spikes did not show plane-wave propagation. In the internal array, we found a total of 35 propagations. In most cases, there were less propagations found in the internal array, compared with the external grid (Table 1).

**Figure:**
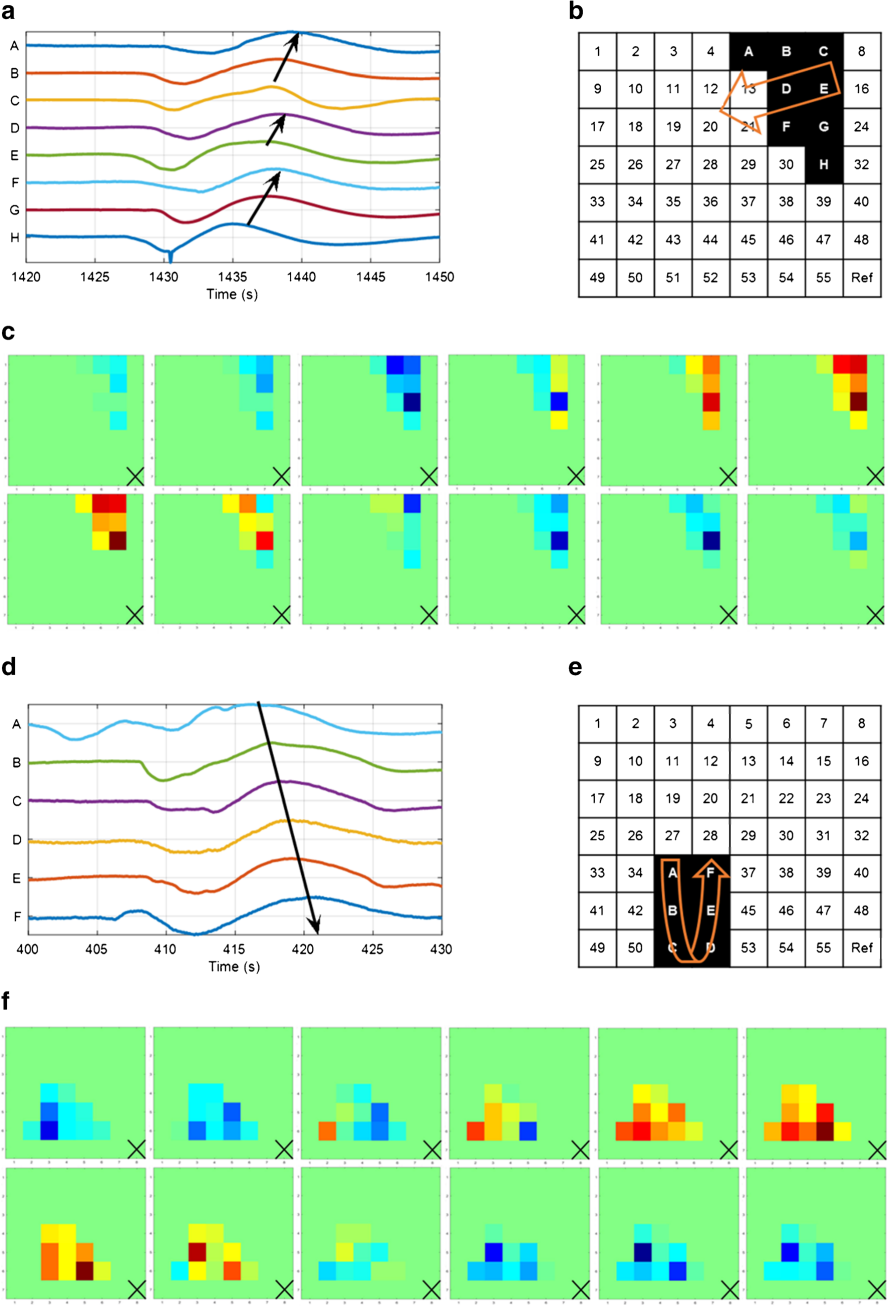

For the 17 events in uterus number 2 showing plane-wave propagation, speed and direction of the conduction velocity vector were estimated using a maximum likelihood approach previously proposed for EHG analysis in pregnancy [34]. This resulted in a median speed of 3.5 mm/s (range 1.6–8.5 mm/s). Concerning the propagation direction, 58.8% of propagation events occurred from left to right, and 41.2% from the fundus to cervix (upper to lower part of the uterus). We measured no event showing propagation right to left or cervix to fundus.

**Fig. 6:**
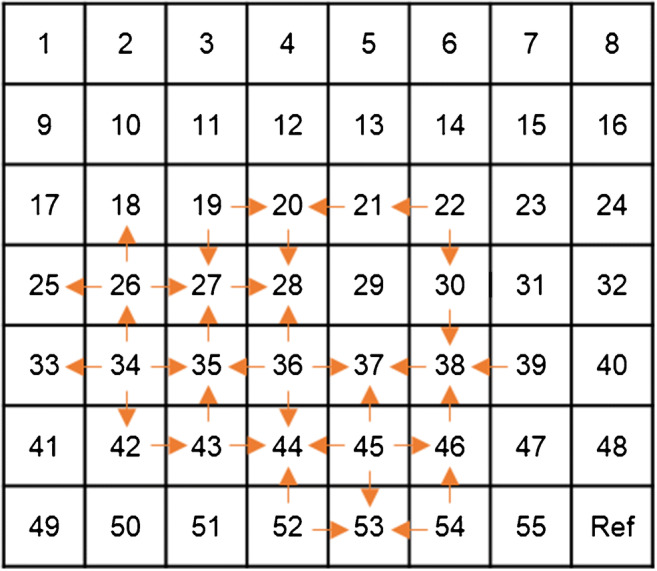
Example of erratic propagation in uterus number three at T0, time interval 200–250 s. The arrows show a simplified projection of the direction of propagation between adjacent electrodes on the external grid. No clear propagation pattern can be observed. See electronic supplementary material for the corresponding video showing propagation in time

**Fig. 7:**
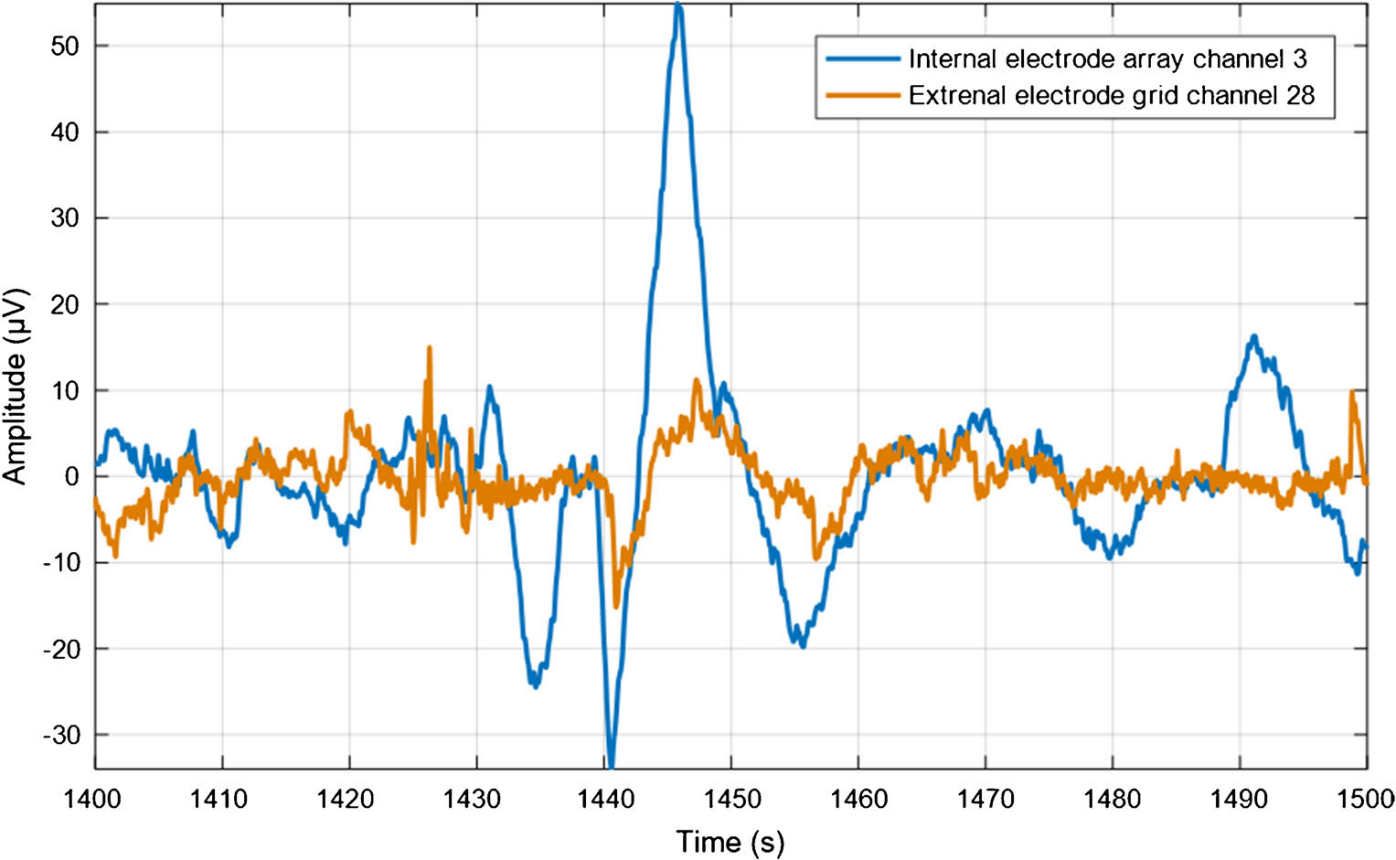
Example of simultaneous recordings of the external grid (orange) and internal array (blue) with comparable shapes

For the events showing erratic propagation, the peak value of every spike was used to estimate the inter-channel delay and define its propagation pattern. In most cases, no clear pattern was found and no delay could be calculated (Fig. 6). In both plane-wave and erratic propagation events, we could not find a preferred pacemaker region and the location from which spikes originated varied over different events. Of the 18 plane-wave propagation events detected externally, 10 also showed propagation on the internal array. Figure 7 shows an example of how the signals of the internal and external recording matched. The speed of the external signals was calculated along the uterine vertical axis and compared with the speed of the internal signals. A median speed equal to 3.4 mm/s was found externally, against a median speed equal to 6.15 mm/s internally. No correlation between the internal and external velocities was found (Pearson’s correlation coefficient = 0.018, *p* = 0.96).

For uterus number 2, which was the only uterus in which plane-wave propagation was dominant, we extended the propagation analysis to *T1* and *T2*. We found that the number of propagation events per minute decreased (*T0* = 0.57/min, *T1* = 0.33/min, *T2* = 0.20/min), but all did show plane-wave patterns, i.e. there was no switch to erratic propagation. The mean speed ranged from 4.0 mm/s at *T0* to 13.6 mm/s at *T1* and 4.6 mm/s at *T2*.

No preferred direction was found at *T1* and *T2*.

### Decay of biopotentials over time

**Fig. 8:**
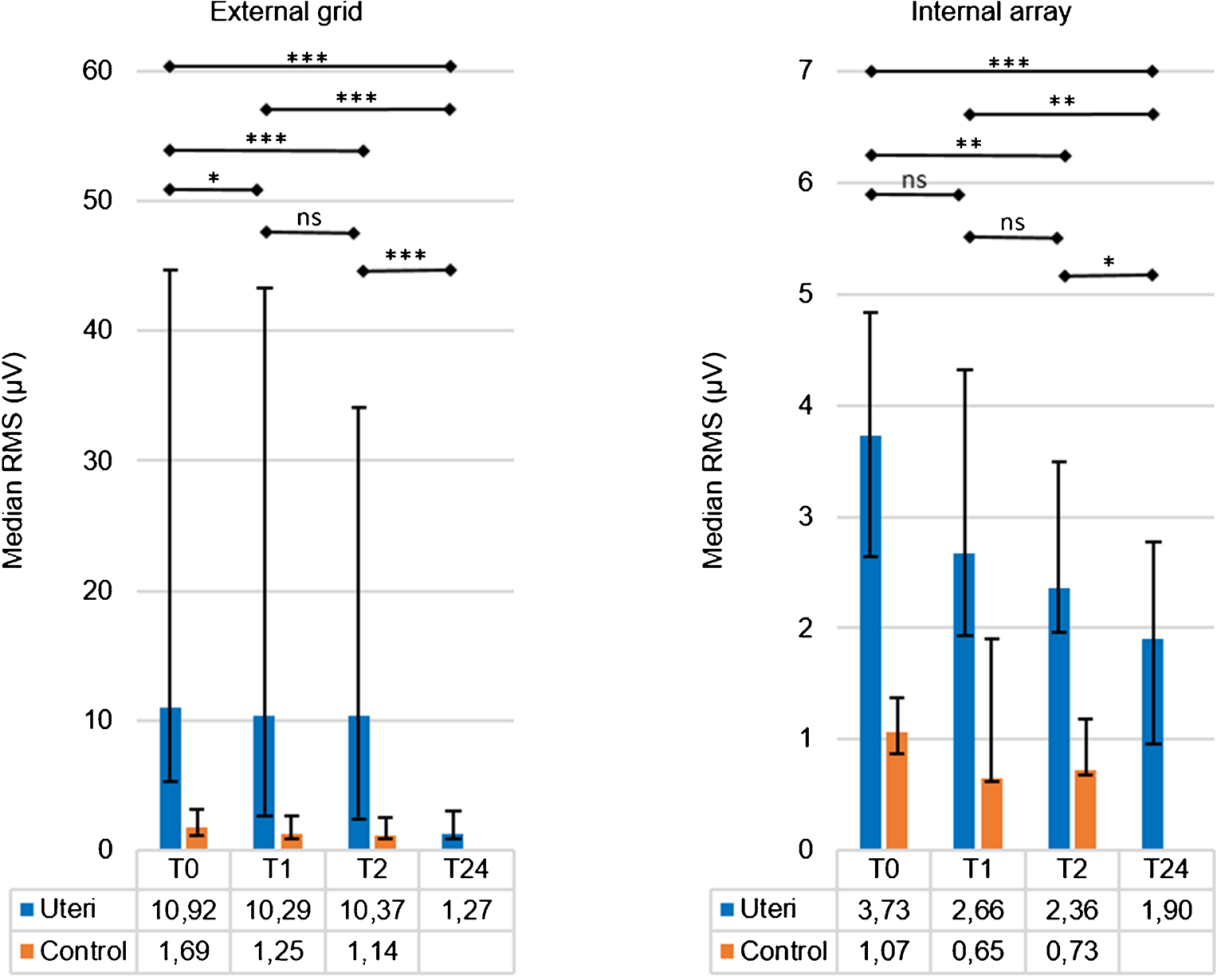
RMS values of all 5 uteri measured directly after surgery (T0) and after 1, 2, and 24 h (T1, T2, and T24, respectively) and of control (chicken breast meat) measured at T0, T1, and T2. The results are expressed as medians of the signals of the external grid (5 uteri times 55 signals) and the internal array (5 uteri times 8 signals). The error bars represent the interquartile range. Statistically significant differences in time were tested using a generalized linear model (SPSS Inc., Chicago, IL, USA): *p < 0.05, **p < 0.01, ***p < 0.001, ns = no significant difference. Although not depicted in this figure, the values at T0, T1, and T2 were significant different from the control (orange bars) in both the external grid and the internal array (Mann Whitney U test: p value at least < 0.05)

Over time, the RMS values showed a decrease in most uteri. The median RMS of all external grid measurements at *T0* was 10.92 μV (IQR 5.35–44.66 μV) and significantly differed from the median at *T1* (10.29 μV, IQR 2.66–43.30 μV), *T2* (10.37, IQR 2.37–34.04 μV), and *T24* (1.27, IQR 0.86–3.04), with *p* values of 0.011, < 0.001, and < 0.001, respectively (Fig. 8). *T1* and *T2* also differed significantly from *T24* (both with *p* values < 0.001). There was no significant difference between *T1* and *T2* (*p* = 0.250). Like at *T0*, the RMS values of all uteri at *T1* and *T2* were significantly different (*p* < 0.05) from control (median of 1.25 μV, IQR 0.91–2.58 μV at *T1* and 1.14 μV, IQR 0.88–2.57 μV at *T2*). At *T24*, the RMS values were not significantly different from control at either *T0*, *T1*, or *T2* (*p* = 0.125, *p* = 0.837, *p* = 0.922, respectively). For the internal array measurements, the median RMS at *T0* was 3.73 μV (IQR 2.63–4.83 μV), which differed significantly from the median at *T2* (2.36 μV, IQR 1.96–3.49 μV, *p* = 0.002) and at *T24* (1.90 μV, IQR 0.95–2.77 μV, *p* < 0.001) but not from the median at *T1* (2.66 μV, IQR 1.93–4.32 μV, *p* = 0.060). Comparable with the external results, the values of both *T1* and *T2* differed significantly from *T24* (*p* = 0.005 and *p* = 0.041, respectively). There was no significant difference between *T1* and *T2* (*p* = 0.226). The RMS values of all uteri at *T1* and *T2* were significantly different (*p* values < 0.05) from control (median 0.65 μV, IQR 0.62–1.90 μV at *T1* and median 0.73 μV, IQR 0.67–1.18 μV at *T2*). At *T24*, the RMS values were not significantly different from control at *T0* and *T1* (*p* = 0.165 and *p* = 0.064, respectively), but they were different from control at *T2* (*p* = 0.011).

## Discussion

To the best of our knowledge, this is the first study investigating the propagation of the biopotentials in ex vivo, non-pregnant human uteri. Based on our results, spontaneous biopotentials can be measured and characterized on an unstimulated ex vivo uterus. This conclusion is supported by two observations: significantly higher average RMS than control showing a decay over time, and the presence of propagation both internally and on the uterine surface.

Remarkable is the variance in RMS values of the external measurements when comparing the 5 included uteri. We do not have one clear explanation for this. A factor which might play a role is the time which the tissue has been deprived of oxygen before starting the measurement. Although we tried to keep conditions as comparable as possible (same sort of procedure, same surgeon, and starting the measurement as quickly as possible after the organ was removed), not every surgical procedure follows exactly the same timeline. We did not record the time between cutting of the blood supply and start of the measurement, so it is impossible to find a correlation there. The way the uterus was handled by the surgeon during procedure could also have some influence on the wetness of the organ, which in turn could have its influence on the electrode-surface contact impendence. Although we tried to optimize the electrode contact using tape and tried to prevent evaporation using the aluminium foil, variations in electrode contact could have caused variations in RMS values. The fact that we lack any prior knowledge on the expected RMS values makes it hard to interpret these complex confounders, which is why in this paper, we provide a direct comparison of the measured RMS values.

Propagation events were found in all patients. Plane-wave propagations were almost exclusively and consistently observed in uterus number 2. Although no general conclusions can be drawn from one patient, the remarkable difference with respect to the other four patients motivated us to report these results, which may bring relevant insight on the different physiological processes taking place in the different phases of the menstrual cycle. Indeed, the only factor that distinguished patient number 2 from the others was the fact that she was menstruating at the time of the surgery. This suggests that the pattern of biopotentials is different in this specific phase of the menstrual cycle. Intra-uterine pressure measurements [5] also showed peculiar properties of uterine contractions during menses as opposed to the other phases of the cycle. During menses, contractions are typically described as ‘labour-like’, with low contraction frequency and high amplitude. In the other phases, ‘peristalsis-like’ activity, with a high contraction frequency and a lower amplitude, is mostly reported [5]. Ultrasound studies suggest the outer two-third of the myometrium to be mostly involved in the contraction during menses [4, 28, 53], while in the other phases, contractions are concentrated in the inner third of the myometrium, the junctional zone [4, 49]. From the electrophysiological point of view, the fact that uterus number 2 was also the only one with a typical burst-like organization of electrical spikes suggests an additional link between menstruation and pregnancy, where slow and cyclic patterns of action potential bursts are typically measured by EHG [35]. Data on propagation direction was only available from uterus number 2, showing a preferred propagation direction from the fundus to the cervix. This seems in agreement with the physiological function of the uterus during menstruation, corresponding to the phase of this uterus at the time of resection. In fact, during menstruation, propagation from the fundus to the cervix can favour down-streaming and emptying of uterine discharge.

A direct comparison of our finding with the literature on non-pregnant uteri is complicated by the fact that previous studies mainly investigated the cine-mechanical properties of the uterus by ultrasound or pressure measurements as opposed to our study that focusses on electrophysiology. While the electrical and mechanical activities of muscles are closely related, a direct relationship for the non-pregnant uterus has not been described yet. Unfortunately, an additional intra-uterine pressure catheter could not be included in our measurement set-up as it would interfere with the internal array. In our preliminary study [42], ultrasound measurements using a transvaginal probe on the external surface of the unperfused ex vivo uterus did not show any spontaneous mechanical activity. Based on the literature, even perfused ex vivo uteri need some stimulations to show mechanical activity [3, 9, 40].

Like in the pregnant uterus [35], we could not find any specific pacemaker region in this study. In the human uterus, ICLCs were found that are similar to the Cajal cells which act as pace making cells in the gastro-intestinal tract [14]. Although histologic studies have shown spontaneous biopotentials in these uterine cells [7] and spontaneous contractions in pregnant uterine tissue samples [33], there is no hard evidence that these cells have the same function as the Cajal cells in the gastro-intestinal tract. The conduction velocities of plane-wave propagation in this study (range 1.6–8.5 mm/s) are in the absolute lower range of what is mentioned in the literature (range 1–520 mm/s) [35] in both humans and animals. However, a direct comparison of speed values with literature is complicated by the fact that the reference figures are based on uteri in the pregnant or even labouring phase. Furthermore, little is known on the effects of organ resection and lack of perfusion on the characteristics of electrical propagation. Only a very weak correlation could be found between the internal and external propagation properties. A certain level of disagreement was evident also in the amplitude of the internal and external signals. This discordance might be partially explained by the complex architecture of the uterus. The myometrium consists of multiple muscle layers with different fibre orientations (and possibly different functions and embryologic origins) [32], which could result in different conduction properties of the electrical activity in the inner and outer layers. The effect of the different geometries, materials, and types of sensors employed internally and externally may also explain the observed weak correlation.

Over time, we measured a decrease in the global amplitude of the biopotentials; this decrease was significant for external measurements between *T0* and *T1*, between *T0* and *T2*, and between *T0* and *T24*, and for the internal measurements between *T0* and *T2* and between *T0* and *T24*. This decrease could be associated to progressive deterioration of the organ tissue. Up to 2 h after resection, we still found a significantly different RMS value relative to control, which we did not find after 24 h. Histological studies, focussing on uterine preservation in a transplantation setting, illustrated that the unpreserved uterus shows severe deterioration after 12 h [2] and that warm ischemia of more than 4 h already makes the uterus unsuitable for transplantation [8, 21]. This suggests that the five measured uteri were most probably ‘electrically dead’ and not able to produce any relevant biopotential after 24 h.

While not diagnosed upfront, in four out of the five included patients, pathologic evaluation of the uterine tissue revealed either adenomyosis or a leiomyoma (Table 1). Both these pathologies can have an influence on uterine contractility, as they seem to disrupt the directed contraction patterns during ovulation [18–20, 24]. These conclusions are based on the mechanical aspects of uterine contractions, observed with either magnetic resonance imaging (MRI) or hysterosalpingoscintigraphy (HSSG). Both pathologies have the ability to interfere with the integrity of the myometrial smooth muscle tissue, which could affect the contraction pattern and propagation, although there are currently no studies supporting this hypothesis. For the validity of this study, it would have been optimal to only include women without any pathology. Unfortunately, it is hard to find women for whom a hysterectomy is indicated without any uterine problem. In our population, the indication was heavy menstrual bleeding or pain at inclusion, for which adenomyosis was diagnosed afterwards. Looking at our results, uterus number 5 (no pathologies) showed RMS values in the same range as the other four, and the number of propagation events did not evidence particular differences, suggesting that the effect of the detected pathologies on our results may be negligible.

To conclude, this study proves that spontaneous biopotentials can be measured in the non-pregnant human uterus ex vivo and suggests that the organ remains electrically active for at least 2 h after surgical resection. In this time frame, electrical propagation can be measured and characterized, possibly providing novel insights into the physiology of this organ. In particular, this study supports the theory that the uterus is an organ with autonomic initiation and, in line with qualitative in vivo studies [5, 15], highlights the peculiarity of the menstrual phase. Obviously, these results are based on an observational study including five uteri only and must be interpreted with caution. Further research with a larger sample size is necessary to draw well-established conclusions. Nevertheless, these results may pave the way for future studies aiming at a better understanding of the complex (electro-)physiology of the non-pregnant uterus, contributing to future diagnostics and treatment involving monitoring and management of uterine contractions, e.g. to improve fecundability.

## Electronic supplementary material

ESM 1 A visual representation of 50 seconds of electrode measurements in patient 2, timeframe 1420-1470 seconds. This example shows plane-wave propagation as shown in figure 5A and B. The coloured vertical bar on the left represents the 8 channels of the internal electrode array and the coloured grid represents the 8x7 external electrode grid.
The colours represent signal amplitude. Red and blue correspond to a positive and negative signal, respectively (range -500 to 500 μV). The channels which do not show any propagation are set to a constant light green colour (0 μV). The black crosses represent electrodes that didn’t work during the measurement. The horizontal graphs show the individual signals of all the electrodes: top left shows the 8 internal array electrodes, the 7 graphs on the right each show 8 signals of the grid, divided by row. (avi 107 MB).

ESM 2 A visual representation of 50 seconds of electrode measurements in patient 3, timeframe 400-450 seconds. This example shows erratic propagation as shown in figure 5C and D.
The coloured vertical bar on the left represents the 8 channels of the internal electrode array and the coloured grid represents the 8x7 external electrode grid. The colours represent signal amplitude. Red and blue correspond to a positive and negative signal, respectively (range -500 to 500 μV). The channels which do not show any propagation are set to a constant light green colour (0 μV). The black crosses represent electrodes that didn’t work during the measurement. The horizontal graphs show the individual signals of all the electrodes: top left shows the 8 internal array electrodes, the 7 graphs on the right each show 8 signals of the grid, divided by row. (avi 104 MB).

ESM 3 A visual representation of 50 seconds of electrode measurements in patient 3, timeframe 200-250 seconds. This example shows erratic propagation as shown in a simplified manner in figure 6.
The coloured vertical bar on the left represents the 8 channels of the internal electrode array and the coloured grid represents the 8x7 external electrode grid. The colours represent signal amplitude. Red and blue correspond to a positive and negative signal, respectively (range -500 to 500 μV). The channels which do not show any propagation are set to a constant light green colour (0 μV). The black crosses represent electrodes that didn’t work during the measurement. The horizontal graphs show the individual signals of all the electrodes: top left shows the 8 internal array electrodes, the 7 graphs on the right each show 8 signals of the grid, divided by row. (avi 111 MB).

## Data Availability

The datasets generated and analyzed during the current study are available from the corresponding author on reasonable request.
